# Measurement of the cavity dispersion in quantum cascade lasers using subthreshold luminescence

**DOI:** 10.1515/nanoph-2025-0078

**Published:** 2025-08-01

**Authors:** Barbara Schneider, Jérôme Faist

**Affiliations:** ETH Zürich, Zürich, Switzerland

**Keywords:** quantum cascade lasers, dispersion, frequency combs

## Abstract

The measurement of the cavity dispersion is a key ingredient in the characterization of a laser frequency comb due to its fundamental role in predicting the multimode laser dynamics. In this work, we discuss the measurement of the modal refractive index dispersion in quantum cascade lasers obtained through the analysis of their sub-threshold luminescence measured using Fourier-transform spectrometers. In particular, we demonstrate the effects of apodization and zero-padding. Finally, we use selected examples to show the effects of different cold-cavity dispersion profiles on the lasing state of the laser. Our results provide a comprehensive guide for determining the cavity dispersion of Fabry–Pérot quantum cascade lasers, which is essential for designing high-performance frequency combs.

## 1 Introduction

The development of laser frequency combs has revolutionized the field of spectroscopy, metrology, and telecommunications [1]. In the mid-infrared spectral region, quantum cascade lasers (QCLs) have emerged as a promising platform for generating frequency combs due to their broad gain bandwidth and high power output [2], [3], [4].

A critical aspect in the design of such combs is the group velocity dispersion (GVD) of the cold cavity, which is a measure of the relative speed at which light travels through a medium with respect to its frequency. The significance of this quantity has been a focus of the field since the first demonstration of comb formation in QCLs [3], [5], as a flat GVD close to zero results in approximately equidistant spacing of the modes supported by the laser cavity itself, which is beneficial for the stability and bandwidth of the spectra thereof. Accordingly, dispersion engineering has long been a focus of research in the field of QCL frequency combs, with a heavy focus on waveguide design [6], [7], [8], [9]. Further developments in the field have highlighted how the GVD significantly influences the laser dynamics as well as their spectral bandwidth and uniformity [3], [5], [10], [11], [12], [13], [14]. Therefore it is crucial to obtain an accurate estimate of the cold-cavity dispersion to further improve both the devices and the modeling thereof.

Accordingly, it is of utmost importance to have a reliable method for characterizing the cold-cavity dispersion of Fabry–Pérot QCLs. Due to the monolithic nature of these devices, as well as required broad spectral probing bandwidth, the most promising approach to this end is the adapted Hakki–Paoli method [15] where the electroluminescence of the laser cavity, measured via a Fourier transform infrared spectrometer, is used to probe its own dispersion [15], [16]. To this end, the amplified spontaneous emission of the laser is coupled into an interferometer, where the interferogram is analyzed to extract the dispersion of the cavity [16]. While it has been used extensively in the past [8], [14], [17], the method has not been analyzed for its accuracy and precision.

In this study, we delve into the method of analyzing sub-threshold interferograms for determining cold-cavity dispersion, emphasizing the influence of preprocessing and zero-padding on the accuracy of the extracted dispersion. Moreover, we show how the measurement of multiple satellites in a single interferogram as well as the measurement at different sub-threshold currents can be used to obtain an estimate of the accuracy of the extracted dispersion. Based on our findings, we provide a comprehensive guide for determining the cold-cavity dispersion of Fabry–Pérot QCLs, which is essential for designing high-performance frequency combs. Additionally, we illustrate the effects of various cold-cavity dispersion profiles on the laser’s lasing state through specific examples. Our results offer a valuable resource for researchers focused on developing high-performance frequency combs with Fabry–Pérot quantum cascade lasers.

## 2 Derivation of the dispersion in a sub-threshold interferogram

**Figure 1:**
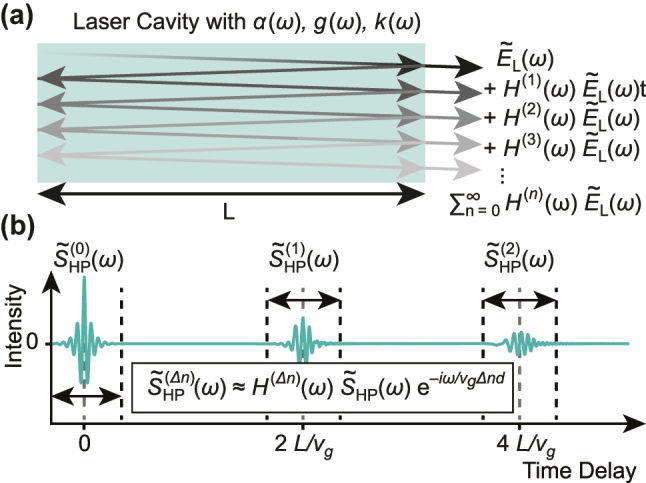
Schematic of the sub-threshold signal generation in a Fabry–Pérot cavity. (a) The path of the light inside the cavity and its relation to the transfer function of the cavity. (b) Qualitative representation of the sub-threshold interferogram of the luminescence inside a Fabry–Pérot cavity.

We consider a Fabry–Pérot semiconductor laser cavity driven below threshold, where the cavity is formed by the gain medium and the cleaved facets act as the mirrors (Figure 1). The field produced by the amplified spontaneous emission, denoted as ${\tilde{E}}_{\text{L}}\left(\omega \right)$ in the frequency domain, with the corresponding power spectral density (PSD) ${\tilde{S}}_{\text{L}}\left(\omega \right)$ , is partially reflected by the facets and thus the total field, ${\tilde{E}}_{\text{ST}}\left(\omega \right)$ , which is emitted from the cavity can be written using the transfer function

(1) $$H\left(\omega \right)=\overset{\infty }{\underset{n=0}{\sum }}{\text{e}}^{\Delta g\left(\omega \right)nd}{\text{e}}^{-\text{i}knd}$$

as

(2) $${\tilde{E}}_{\text{ST}}\left(\omega \right)=H\left(\omega \right){\tilde{E}}_{\text{L}}\left(\omega \right),$$

where *g*(*ω*) − *α*(*ω*) = Δ*g*(*ω*) is the averaged net gain, 2*L* = *d* is the roundtrip length of a Fabry–Pérot cavity with length *L*, and *k* is the wave number. When we couple this light into an interferometer, we obtain an interferogram that can in turn be written as

(3) $$\begin{array}{ll} S_{\text{ST}}\left(\tau \right) & =\overset{\infty }{\underset{m,n=0}{\sum }}F^{-1}\left\{{\tilde{S}}_{\text{L}}\left(\omega \right){\text{e}}^{\Delta g\left(\omega \right)\left(m+n\right)d}{\text{e}}^{-\text{i}k\left(m-n\right)d}\right\}\\ & =\overset{\infty }{\underset{\Delta n,n=0}{\sum }}F^{-1}\left\{{\tilde{S}}_{\text{L}}\left(\omega \right){\text{e}}^{\Delta g\left(\omega \right)\left(2n+\Delta n\right)d}{\text{e}}^{-\text{i}k\Delta nd}\right\}\\ & \;+\overset{\infty }{\underset{\Delta n,n=0}{\sum }}F^{-1}\left\{{\tilde{S}}_{\text{L}}\left(\omega \right){\text{e}}^{\Delta g\left(\omega \right)\left(2n-\Delta n\right)d}{\text{e}}^{\text{i}k\Delta nd}\right\}\\ & =\overset{\infty }{\underset{\Delta n=0}{\sum }}F^{-1}\left\{\frac{{\tilde{S}}_{\text{L}}\left(\omega \right)}{1-{\text{e}}^{2\Delta g\left(\omega \right)d}}{\text{e}}^{\Delta g\left(\omega \right)\Delta nd}{\text{e}}^{-\text{i}k\Delta nd}\right\}\\ & \;+\text{c.c.}, \end{array}$$

where the double sum over *m*, *n* can be simplified into a single sum over Δ*n*, which is the difference in the number of roundtrips between the components of the interfering signals, by inserting the solution to the sum over *n* for e^2Δ*g*(*ω*)*dn*^ with negative net gain *g*(*ω*). $F^{-1}$ denotes the inverse Fourier transform. This expression can therefore be viewed as a superposition of sub-interferograms corresponding to any difference in the number of roundtrips Δ*n* within the cavity. Correspondingly, the isolated interferogram of the Δ*n*-th satellite can be written as

(4) $$S_{\text{ST}}^{\left(\Delta n\right)}\left(\tau \right)=F^{-1}\left\{\frac{{\tilde{S}}_{\text{L}}\left(\omega \right)}{1-{\text{e}}^{2\Delta g\left(\omega \right)d}}{\text{e}}^{\Delta g\left(\omega \right)\Delta nd}{\text{e}}^{-\text{i}k\Delta nd}\right\}+\text{c.c.}$$

Expanding the wave number *k*(*ω*) around the center frequency *ω*_0_ as

(5) $$k\left(\omega \right)=k_{0}+\frac{\left(\omega -\omega_{0}\right)}{v_{g}}+D_{\text{int}}\left(\omega ,\omega_{0}\right),$$

where *k*_0_ is the wave number at the center frequency *ω*_0_, *v*_*g*_ is the group velocity at *ω*_0_, and *D*_int_(*ω*, *ω*_0_) is the integrated dispersion related to the GVD as

(6) $$\frac{\partial^{2}D_{\text{int}}\left(\omega ,\omega_{0}\right)}{\partial \omega^{2}}=\frac{\partial^{2}k\left(\omega \right)}{\partial \omega^{2}}=D_{g}\left(\omega \right).$$

By using this definition, we do not just restrict ourselves to the special case where the dispersion is constant across the optical spectrum, but also take into account the existence of higher-order terms. We can rewrite the interferogram as

(7) $$\begin{array}{ll} S_{\text{ST}}^{\left(\Delta n\right)}\left(\tau \right) & =S_{\text{L}}\left(\tau \right)*F^{-1}\left\{\frac{{\text{e}}^{\Delta g\left(\omega \right)\Delta nd}}{1-{\text{e}}^{2\Delta g\left(\omega \right)d}}{\text{e}}^{-\text{i}D_{\text{int}}\left(\omega ,\omega_{0}\right)\Delta nd}\right\}\\ & \;*\delta \left(\tau -\frac{d\Delta n}{v_{g}}\right)+\text{c.c.}, \end{array}$$

with *S*_L_(*τ*) being the interferogram of the luminescence signal without the cavity, and *δ* being the Dirac delta function. The operator ∗ denotes the convolution operation. In this form we can see that the interferogram is a sum of shifted versions of the interferogram of the luminescence signal, convolved with the net gain contribution and a phase term induced by then GVD of the cavity.

When the width of the luminescence spectrum is much wider than the free spectral range of the cavity, we can assume negligible overlap of the shifted interferograms for small Δ*n* and any single satellite can be isolated by cropping the interferogram around its center as

(8) $$\begin{array}{ll} S_{\text{HP}}^{\left(\Delta n\right)}\left(\tau \right) & =A\left(\tau \right)S_{\text{L}}\left(\tau \right)*F^{-1}\left\{\frac{{\text{e}}^{\Delta g\left(\omega \right)\Delta nd}}{1-{\text{e}}^{2\Delta g\left(\omega \right)d}}{\text{e}}^{-\text{i}D_{\text{int}}\left(\omega ,\omega_{0}\right)\Delta nd}\right\}\\ & \;+\text{c.c.}, \end{array}$$

where *A*(*τ*) is the apodization function. The Fourier transform of the interferogram is then given by

(9) $${\tilde{S}}_{\text{HP}}^{\left(\Delta n\right)}\left(\omega \right)=\tilde{A}\left(\omega \right)*\left(\frac{{\text{e}}^{\Delta g\left(\omega \right)\Delta nd}}{1-{\text{e}}^{2\Delta g\left(\omega \right)d}}{\tilde{S}}_{\text{L}}\left(\omega \right){\text{e}}^{-\text{i}D_{\text{int}}\left(\omega ,\omega_{0}\right)\Delta nd}\right),$$

with $\tilde{A}\left(\omega \right)$ being the Fourier transform of the apodization function. Since the apodization function and its Fourier-transform are purely real functions, conceptually it does not have an effect on the extracted phase of the satellite. Therefore, using the relationship in Equation (5), we can directly extract calculate the GVD from the complex spectrum of the satellite:

(10) $$D_{g}\left(\omega \right)=-\frac{1}{\Delta n2L}\frac{\partial^{2}\arg\left({\tilde{S}}_{\text{HP}}^{\left(\Delta n\right)}\left(\omega \right)\right)}{\partial \omega^{2}},$$

where *L* is the cavity length.

## 3 Analysis of the experimental sub-threshold interferogram

**Figure 2:**
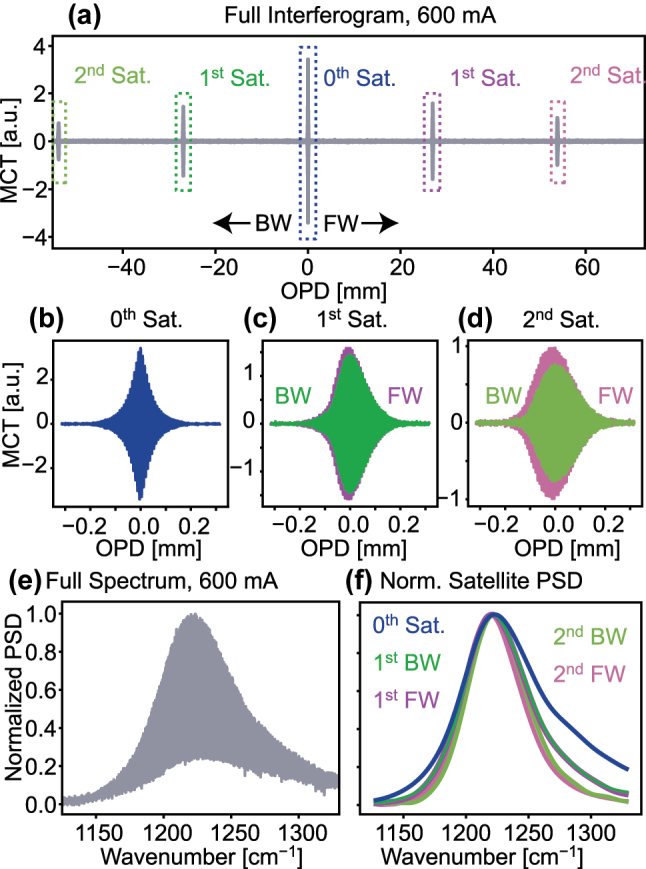
Experimental measurement of the sub-threshold interferogram of a Fabry–Pérot QCL cavity. (a) A double-sided interferogram including the first two satellites on both sides of the central peak, in forward (FW) and backward (BW) direction. (b)–(d) The isolated interferograms of the center peak and the first two satellites. The *x*-axis of the BW satellites is flipped for comparability. (e) The PSD calculated from the interferogram. (f) The normalized PSD of the isolated satellites.

To experimentally measure the GVD of a Fabry–Pérot QCL cavity, we first measure the sub-threshold interferogram of the cavity either directly in an AC-coupled configuration or with the DC-offset removed in post-processing to avoid artifacts. To do so, we can use any interferometer with a resolution higher than approximately 1.5 the free spectral range of the cavity, if we want to include the center peak in the interferogram. The resolution may be even lower, if we only scan across a single satellite. In Figure 2, we show the experimental measurement of the sub-threshold interferogram of a Fabry–Pérot QCL cavity, which has been extensively characterized for its operation as an optical frequency comb [14], [17], [18] which we will refer to as EV2549D in this work, at 600 mA, which is 60 mA below threshold. Here, we have used a double-sided interferogram to include the first two satellites on both sides of the central peak, as is shown in full in Figure 2(a). The PSD calculated directly from this interferogram is shown in Figure 2(e). Since we are interested in the isolated satellites, we can preprocess the data by determining the maxima of the satellite envelopes and cropping the interferogram around these maxima. The isolated interferograms of the center peak and the first two satellites are shown in Figure 2(b)–(d). The visible difference between the forward and backward satellites in the direct comparison in Figure 2(c) and (d) is most likely caused by an imperfect beam-shape, asymmetries in the instrument as well as slight misalignment in the optical path. Accordingly, we can calculate the normalized PSD of each of the isolated satellites, as shown in Figure 2(f). From the PSD of the isolated satellites, the net gain can be extracted using a simple division, as has been extensively discussed in previous works as a diagnostic means for the active region and waveguide design of QCLs [16], [19].

While for the extraction of the net gain the accuracy of the absolute value of the Fourier-transform is the most important, for the extraction of the GVD, the accuracy of the phase is crucial. Since the effects of noise and data processing are not as apparent in the phase as they are in the amplitude, in the following we will elucidate the steps taken to ensure the accuracy of the phase extraction in the GVD measurement.

### 3.1 Satellite width

**Figure 3:**
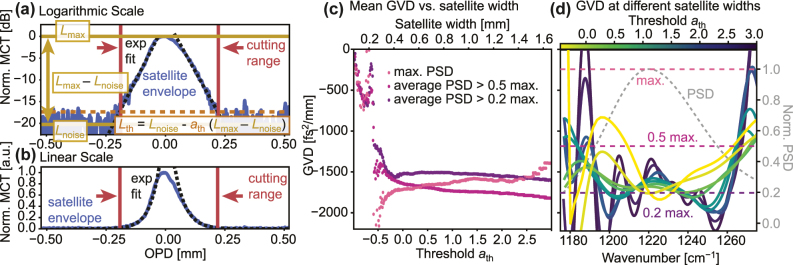
Fitting and width determination of the envelope of the 1st satellite interferogram forward of EV2549D at 600 mA and its effects. (a) The envelope plotted in a logarithmic scale with the exponential fits of the tails plotted as black dashed lines and the noise level *L*_noise_, the maximum of the envelope *L*_max_ as well as their spacing marked in yellow. The cutting threshold is marked in orange and the corresponding cut positions are marked in red. (b) The same envelope plotted in a linear scale with the exponential fits as black dashed lines and the cutting positions marked in red. (c) The extracted GVD as a function of the cutting threshold, using the GVD at the maximum position of the PSD, the average over the range where the PSD is above half of the maximum, and the average over the range where the PSD is above 0.2 of the maximum. (d) The wavenumber-dependent extracted GVD as a function of the cutting threshold together with the normalized PSD of the satellite at one of the cutting thresholds for visual reference.

The first step in the data processing is the isolation of the satellites. In the presence of non-zero GVD in the cavity, we cannot assume that the maximum of the satellite interferogram coincides with the envelope maximum. Therefore, we use the Hilbert transform to calculate the envelope of the satellite and use the maximum of the result as the center of the satellite. Further, for most common gain profiles, we can assume a Lorentzian luminescence spectrum, which allows us to approximate the sides of any satellite envelope as exponentially decaying functions. This allows us to use simple exponential fits to extrapolate the envelope tails below the noise floor, which allows for the isolation of the signal based on its signal-to-noise ratio. With this, small asymmetries in the satellites coming from experimental imperfections can be accounted for as well. In Figure 3(a) and (b), we show the envelope of the first satellite forward of EV2549D at 600 mA, with the exponential fits of the tails plotted as black dashed lines in logarithmic and linear scale. In the logarithmic plot in (a), we can see that the slope of the satellite tails is indeed approximately linear. Here, we can also see the noise level *L*_noise_, calculated from the mean value of the envelope in the linear scale in the region where no signal is present. We find that since the tails are linear in logarithmic scale and therefore their distance grows linearly with the cutting threshold *L*_th_, we can define the quantity

(11) $$a_{\text{th}}=\frac{L_{\text{th}}-L_{\text{noise}}}{L_{\max}-L_{\text{noise}}},$$

which scales linearly with the satellite width and can be used as a general metric for the width of the satellite. In the linear plot in (b), we can see the exponential fits and the cutting positions marked in red. The noise floor is not shown here since it is barely visible in the linear scale. Here, we can again see that the exponential fits are a good approximation of the envelope tails.

Due to the limited width of the satellites, we want to maximize the information content of the data by optimizing the width of the interferogram section we use for the GVD extraction. To do so, we can use the metric *a*_th_ to determine the optimal width of the satellite with respect to the noise level in the data. In the following, we will use this metric to determine the optimal width of the satellite for the GVD extraction.

In Figure 3(c) and (d), we show the effect of using different cutting thresholds, and accordingly, cutting widths. In (c), we show the width-dependence of the GVD, where we compare the GVD at the position of the PSD maximum, and the averaged GVD in the ranges where the PSD is larger than 0.2 and 0.5 its maximum value, respectively. We can see that the width-dependence is very similar for all three metrics, with severely underestimated absolute values below *a*_th_ = −0.5, which corresponds to a threshold of half the distance between the envelope maximum and the noise level in the logarithmic scale. For values of *a*_th_ well above 0, the GVD stabilizes with a slight drift. This corresponds to cutting the satellites where the tails are well below the noise level. The stabilized GVD starts approximately at *a*_th_ = 0, which corresponds to cutting the satellite at the noise level. This intuitively makes sense, since this is the point where any further added information in the data is below the noise level and therefore contributes less than the noise. In (d), we show the wavenumber-dependent GVD calculated at different *a*_th_ > −0.5. In this plot, we can see that in accordance with the increasing nominal resolution of the data, the feature size of the GVD decreases and intermittently converges to a smooth curve with little modulation in the range where the normalized PSD is above 0.5. Starting at $\approx a_{\text{th}}>2.0$ , the GVD starts developing small modulation features which shrink with increasing *a*_th_. This can be attributed to the accumulated noise in the data, which starts to dominate the extracted GVD at this point. Therefore, we can conclude that the optimal width of the satellite for the GVD extraction is around *a*_th_ = 0, which corresponds to cutting the satellite at the noise level.

### 3.2 Apodization

**Figure 4:**
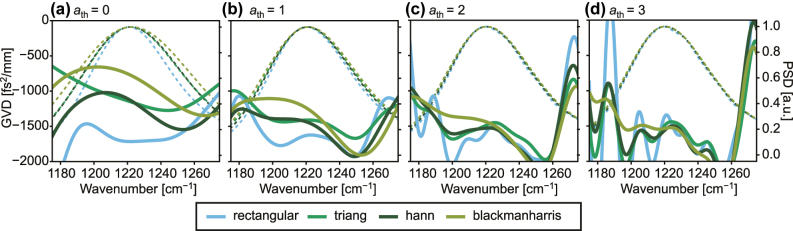
The effect of using apodization windows on the satellite during processing on the extracted wavenumber-dependent GVD of the 1st satellite forward of EV2549D at 600 mA. Comparison of the effects of using the rectangular, the triangular, the Hann and the Blackman–Harris window when (a) *a*_th_ = 0, (b) *a*_th_ = 1, (c) *a*_th_ = 2, and (d) *a*_th_ = 3, together with the respective normalized PSDs of the satellite. The solid lines denote the GVD, while the dashed lines denote the PSD calculated with the different apodizations, which in turn are differentiated in color.

When processing interferograms, the use of apodization windows is a common practice to reduce spectral leakage and improve the signal-to-noise ratio. The key feature of such apodization windows is that they reduce the amplitude of the signal towards the edges of the interferogram. The trade-off is that the apodization window also reduces the width of the signal, which leads to a reduction in frequency-resolution. This is especially critical, when a significant portion of the information in the data is located at the edges of the interferogram, as in the satellites of the sub-threshold interferogram, where the chirp is distributed across the entire satellite. Figure 4 shows the results of calculating both, the GVD (solid lines) and the PSD (dashed lines) using different apodizations and different interferogram widths. In Figure 4(a), we can immediately see the effect of this loss in resolution in that the GVD calculated without a window is significantly further from zero than that calculated with the triangular, Hann or the Blackman–Harris window, when the satellite is cropped where it crosses the noise level. This is further reinforced by the difference in width of the respective PSDs. As the width of the cut interferogram section is multiplied in Figure 4(b)–(d), the GVD calculated with and without apodization windows converges. However, at these widths, it becomes increasingly difficult to discern the effects of the noise in the data from the effects of the apodization window, as we can see similar oscillatory features arising in all curves. Therefore, we can conclude that the use of apodization windows is not advisable when the focus of the measurement is the phase information to avoid loss of accuracy in the GVD extraction.

### 3.3 Zero-padding

**Figure 5:**
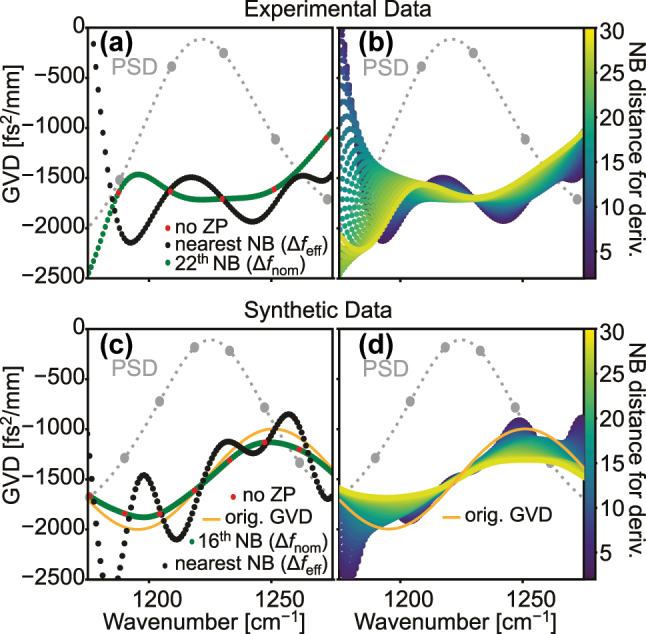
The effect of zero-padding on the extracted wavenumber-dependent GVD of the 1st satellite forward. (a) The GVD extracted from the experimental data of EV2549D at 600 mA without zero-padding (red dots), where the spacing between the dots corresponds to the nominal resolution given by the length of the interferogram, Δ*f*_nom_. The result with zero-padding and calculating the discrete derivative using the nearest neighboring data points (black dots), where the spacing between the dots corresponds to the effective resolution, Δ*f*_eff_, given by the combined length of the interferogram and the zero-padding, and with zero-padding and calculating the discrete derivative using the data points spaced like the original data (green dots), where the spacing between the dots corresponds again to the nominal resolution, Δ*f*_nom_, given only by the length of the interferogram. (b) Comparison of the wavenumber-dependent GVD calculated with the discrete derivative using the *n*th nearest neighboring data points. (c) Synthetic data generated as an approximation of the experimental data using the formulas introduced in Section 2 and plotted in analogy to (a). (d) The results from using the synthetic data to generate the same comparison as shown in (b). The imposed GVD is added in dark yellow for reference.

Another aspect of data processing is the use of zero-padding to increase the resolution of the Fourier-transform. This is especially necessary when the length of the interferogram is small and therefore the nominal resolution of the data, Δ*f*_nom_ = Δ*τ* ^−1^, where Δ*τ* is the length of the transformed interferogram, is low. The addition of zeros at one end of the interferogram leads to an effective interpolation of the Fourier-transformed data. This, while not increasing the information content of the data, is an important measure to prevent undersampling of the transformed data and accordingly, by enabling a much smaller effective data point spacing, Δ*f*_eff_, increase the accuracy of the extracted information. While the use of zero-padding is generally advisable, it needs to be applied with caution when the results are to be processed further. In our case, the GVD is obtained by calculating a second derivative of the phase of the Fourier-transformed data, which is a highly sensitive operation. When working with discrete data, the derivative is generally calculated using the difference quotient between the nearest neighbors. When these nearest neighbors, however, are the result of interpolation, there is no general rule, that their difference quotient matches that of the nominally nearest neighbors. Therefore, as shown in Figure 5, we can find that the use of zero-padding can lead to significant artifacts in the extracted GVD. In (a), we can immediately see by comparing the GVD calculated without zero-padding (red), with the neighbors spaced by Δ*f*_nom_, and that calculated from the zero-padded data without regard to its effects (black), with the neighbors spaced by Δ*f*_eff_, that there is a non-negligible difference between the two. This can be understood by taking into account the fact that the datapoints added by zero-padding are not real data points, but rather interpolated data points. Therefore, the use of zero-padding can lead to significant artifacts in the extracted GVD. This can be avoided by calculating the second derivative not from adjacent datapoints but by viewing the transformed zero-padded data as multiplexed shifted versions of the unpadded data, calculating the second derivative from the demultiplexed versions and remultiplexing the results. This is shown in green in (a). In (b), we can see how the calculcating the second derivative from the *n*th nearest neighboring data points changes the shape of the GVD. When using a spacing much smaller than the original data, the GVD is dominated by oscillatory features with a period corresponding to the nominal resolution of the data. With increasing spacing, this is smoothed out and the GVD progressively loses features, as would be expected since using a wider point spacing for the derivative calculation reduces the effective resolution of the data. In Figure 5(c) and (d), we use synthetic data generated using the expressions previously introduced in Section 2, with a PSD, average GVD and white noise contribution approximating that of the experimental data shown in (a) and (b) and plot it in the same fashion to verify our findings. The imposed dispersion is plotted in dark yellow in both plots and features well-defined higher-order contributions. The comparison with the results after processing the data both highlights once more how choosing the nominal resolution for calculating the second derivative leads to the highest accuracy of the extracted GVD, making this method suited for not only approximating the average GVD but also some of its spectral dependence. In conclusion, zero-padding is an important tool to optimize the precision of the processed data, but the derivative calculation needs to be adapted to the zero-padded data to avoid artifacts in the extracted information.

### 3.4 The ideal data processing chain

In conclusion, the ideal data processing chain for the extraction of the GVD from the sub-threshold interferogram of a Fabry–Pérot QCL cavity is as follows:

1. Approximate the envelope of the satellite using the Hilbert transform and fit the tails of the envelope with exponential functions to extrapolate the envelope below the noise level.
2. Cut the satellite at the noise level to maximize the signal-to-noise ratio.
3. Use zero-padding to increase the resolution of the Fourier-transformed data, but calculate the second derivative from the demultiplexed data to avoid artifacts in the extracted GVD.

We can see that therefore the highest achievable nominal resolution of the data is directly determined by the signal-to-noise ratio of the interferogram. Therefore, its optimization is crucial for the accuracy of the extracted GVD. The use of apodization windows is not advisable, since they disproportionally attenuate the tails of the satellite as well as reduce the effective width of the signal and therefore introduce systematic errors in the extracted GVD.

## 4 Statistics of the GVD extraction

When measuring the sub-threshold interferograms for the GVD extraction, the measurement typically does not only comprise a single satellite but multiple satellites of increasing order. Moreover, there is no one single sub-threshold current that is generally used for the GVD extraction, but rather a range of currents below threshold is used to ensure that any effects of measuring too close to threshold as well as the decrease in signal-to-noise with decreasing current are accounted for. These multiple satellites are convenient as a means to further quantify the accuracy of the GVD extraction. In the following, we will show the effects of using different satellites in a single interferogram as well as the data measured at different currents on the extracted GVD and how this can be used to quantify the accuracy of our results.

**Figure 6:**
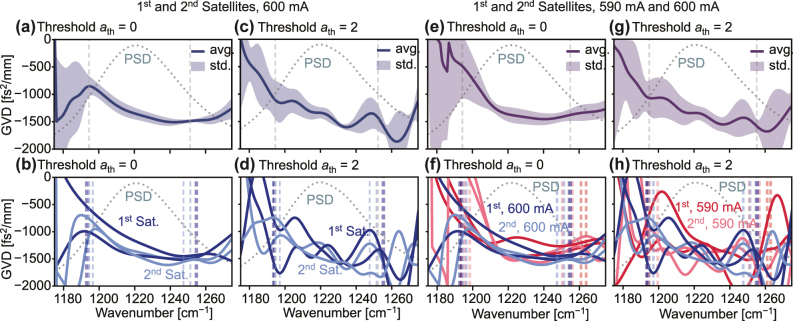
The effect of using different satellites in the GVD extraction of the 1st satellite forward of EV2549D at 600 mA. (a) The average GVD calculated from the 1st and 2nd satellite in the interferogram in both forward and backward direction with the standard deviation marked by the shaded area, when cutting the satellite where its tails enter the noise level and, (b) the corresponding individual traces. (c) and (d) The same, but calculated from the satellites cut at a wider width. (e) The average GVD calculated at 600 mA and 590 mA in both forward and backward direction with the standard deviation marked by the shaded area cut at the same width as in (a) and (f), the corresponding individual traces. (c) and (d) The same as in (e) and (f), but calculated from the satellites cut at a wider width. The average PSD of the satellites is shown for reference together with vertical lines depicting the range where the normalized PSD is above 0.5 for each depicted dataset. The average PSD of the satellites is shown for reference together with vertical lines depicting the range where the normalized PSD is above 0.5 for each depicted dataset.

In the case of the double-sided interferogram depicted in Figure 2(a) for EV2549D at 600 mA, there are two first order and 2 s order satellites available for comparing the data within a single dataset. When applying the same algorithm to all satellites and taking into account the reversed direction of the optical path delay for the backward satellites, we arrive at the results shown in Figure 6(a)–(d). For reference, we show both, the datasets obtained when cutting the satellites where their tails enter the noise level and when drastically increasing the cutting range. In (a) and (b), we can see that the error is relatively low in the range where the normalized PSD of the satellites is above 0.5. When the cutting range is increased, the error increases significantly, which is expected since the signal-to-noise ratio of the data is reduced. In (c) and (d), by comparing the individual datasets, we can see that when cutting at the noise level, the shape of the GVD is relatively stable between the different satellites. However, when cutting at three times the width, the oscillatory features in the GVD show a significant difference between the datasets, which is a strong indication that, indeed, these features are caused by a reduction in the signal-to-noise ratio of the data. With this, we can see that indeed, the different satellites in the interferogram can be used to obtain an estimate of the accuracy of the GVD extraction.

In the same manner, the measurements at different sub-threshold currents can be used to further increase the total number of satellites to be used for statistics and error estimation. In Figure 6(e)–(h), we expand on the plots shown in Figure 6(a)–(d) by adding the analogous satellites measured at 590 mA. In (e) and (f), we can see that the error range has increased slightly, especially toward the edges, where the normalized PSD is below 0.5. In (g) and (h), by comparing again the individual datasets, we can see that the GVD obtained from the satellites at lower currents is comparatively more noise than that at 600 mA, but that again, the overall shape and position is in agreement and does not show any systematic differences. This is a strong indication that the GVD extraction is robust against changes in the sub-threshold current and therefore the choice of the current is not critical for the accuracy of the GVD extraction, but rather for the signal-to-noise ratio of the data.

## 5 Dispersion measurements

**Figure 7:**
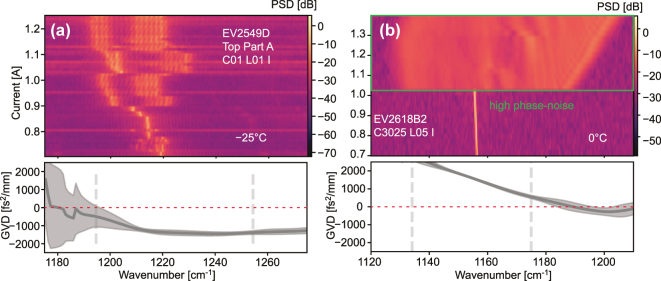
Current-dependent spectra of four different QCLs and the corresponding GVD extracted below threshold. (a) The spectral map above threshold of EV2549D (top part A, C01 L01 I) and the corresponding GVD measured at 590 and 600 mA. (b) The spectral map above threshold of EV2618B2 (C3025 L05 I) and the corresponding GVD measured at 405, 410, 415, 420 and 425 mA. The grey shaded area marks the error range of the GVD extraction, the red dashed line indicates where the GVD is zero, the grey dashed lines indicate where the average electroluminescence spectrum of the satellites drops below half its maximum, and the yellow frame marks the range where EV2618B2 has been found to operate in a high phase-noise state.

Finally, we can use the guidelines we have defined in the previous sections on different datasets to estimate the cold-cavity GVD of different Fabry–Pérot QCL cavities. Firstly, the device EV2549D, which we have used in the previous sections, is shown in Figure 7(a). In the spectral map, we can see that the device lases as a frequency comb throughout a large current range, albeit with limited spectral bandwidth. The corresponding GVD is found to be strictly negative, below −1,000 fs^2^/mm, but comparatively flat. The error range becomes significant where the electroluminescence spectrum of the satellites drops below half its maximum, which can be attributed to the reduced signal-to-noise ratio of the data. For comparison, the device EV2618B2 is shown in Figure 7(b). Here, the spectral map shows that the device lases in quasi single-mode operation at low currents and then directly switches to high phase-noise multimode operation with a very broad spectrum at high currents. The GVD of this device is found to be positive with a strong slope throughout the range where the luminescence spectrum of the subthreshold satellites is above half its maximum. Finally, the device EV2394C is shown in Figure 7(d). The spectral map measured for this device shows a very narrow spectrum throughout the entire current range, with a jump in the spectrum at high currents. The GVD of this device is found to be close to zero with barely any slope where the luminescence spectrum of the satellites is above half its maximum.

By comparing the lasing spectra of the two devices we can immediately see some of the effects of the respective GVD profiles. The flat non-zero GVD of EV2549D results in a limited spectral bandwidth, but nonetheless favorable properties in terms of the early onset of multi-mode operation as well as a high degree of coherence. While the distance from zero can be understood as a cause for the limit in the spectral bandwidth. On the other hand, EV2618B2, with a late onset of multimode operation and low coherence during broadband operation has a significant slope in the extracted GVD. The much larger bandwidth in the latter case can be viewed as the device lasing across almost its entire available gain bandwidth as a result of the loss in coherence, since the equidistance requirement is thus lifted.

## 6 Conclusions

In this work, we have presented a comprehensive analysis of the method for determining the cavity dispersion of Fabry–Pérot quantum cascade lasers using sub-threshold interferograms. We have discussed the theoretical background of the method and provided a detailed guide for the data processing steps necessary to ensure the accuracy of the extracted group velocity dispersion (GVD). Our analysis shows that the optimal data processing chain involves isolating the satellites at the noise level, avoiding the use of apodization windows, and carefully handling zero-padding to prevent artifacts in the GVD extraction. We have demonstrated the robustness of the method by comparing the GVD extracted from different satellites and at different sub-threshold currents, and we have applied the method to several QCL devices to illustrate its practical applicability. Our results provide valuable insights into the dispersion properties of QCLs and offer a useful tool for designing high-performance frequency combs.
